# Vaspin Ameliorates Cardiac Remodeling by Suppressing Phosphoinositide 3-Kinase/Protein Kinase B Pathway to Improve Oxidative Stress in Heart Failure Rats

**DOI:** 10.1097/FJC.0000000000001291

**Published:** 2022-05-03

**Authors:** Mingyue Ji, Yong Li, Yun Liu, Genshan Ma

**Affiliations:** * Department of Cardiology, Zhongda Hospital, Medical School of Southeast University, Nanjing, China;; † Department of Cardiology, Lianshui County People's Hospital, Huai'an, China;; ‡ Departments of Cardiology; and; § Intensive Care Medicine, The First Affiliated Hospital of Nanjing Medical University, Nanjing, China.

**Keywords:** vaspin, heart failure, cardiac remodeling, oxidative stress, PI3K/Akt

## Abstract

This study aimed to explore whether vaspin could alleviate cardiac remodeling through attenuating oxidative stress in heart failure rats and to determine the associated signaling pathway. Cardiac remodeling was induced by myocardial infarction, transverse aortic constriction, or angiotensin (Ang) II infusion in vivo, and the neonatal rat cardiomyocytes (NRCMs) and neonatal rat cardiac fibroblasts (NRCFs) were treated with Ang II. Vaspin treatment alleviated fibrosis in myocardial infarction, transverse aortic constriction, and Ang II-treated rats. The Ang II-induced increases of atrial natriuretic peptide and brain natriuretic peptide in NRCMs and Ang II-induced increases of collagen I and collagen III in NRCFs were reduced after vaspin treatment. Vaspin administration inhibited the Ang II-induced increases of phosphoinositide 3-kinase/protein kinase B (PI3K/Akt) pathway, superoxide anions, malondialdehyde, and NADPH oxidases activity in NRCMs and NRCFs. The overexpression of PI3K, Akt, or NADPH oxidases 1 reversed the attenuating effects of vaspin on Ang II-induced elevation of atrial natriuretic peptide and brain natriuretic peptide in NRCMs, as well as Ang II-induced increases of collagen I and collagen III in NRCFs. The administration of wortmannin (PI3K inhibitor) or MK2206 (Akt inhibitor) inhibited the oxidative stress induced by Ang II in NRCMs and NRCFs. The above results suggest that vaspin can alleviate cardiac dysfunction and remodeling in heart failure rats. Vaspin attenuates Ang II-induced hypertrophy of NRCMs and fibrosis of NRCFs through suppressing PI3K/Akt pathway to alleviate oxidative stress.

## INTRODUCTION

Cardiovascular diseases are major causes of death globally.^1^ Cardiac remodeling, a pathophysiologic process in cardiovascular diseases, has garnered considerable attention. Cardiac remodeling occurs after volume overload, high pressure, or ischemic injury during the development of heart failure.^2^ Cardiac hypertrophy and fibrosis, 2 essential changes in cardiac remodeling, are key to the initiation and progression of some cardiovascular diseases.^3^ Myocardial hypertrophy features rising heart weight and the activation of genes including atrial natriuretic peptide (ANP) and brain natriuretic peptide (BNP).^4,5^ Cardiac hypertrophy usually results in fibrosis, which is manifested by collagen type I and collagen type III accumulation.^6^

Vaspin, first identified in 2005, is an inhibitor of visceral adipose tissue–derived serine protease and belongs to the serine protease inhibitor family.^7^ Serum vaspin is an important prognostic marker of major adverse cardiac events in acute myocardial infarction (MI).^8^ The plasma vaspin concentrations of patients with major adverse major cardiac events were lower than those without these events.^9^ Vaspin treatment can alleviate diabetes-induced myocardial injury and exert a cardioprotective effect.^10^ Yet, the regulatory role of vaspin in cardiac remodeling of heart failure remains unclear.

Akt is activated in the angiotensin (Ang) II-induced fibrotic cardiac fibroblasts.^11^ Significant upregulation of protein expression of phosphoinositide 3-kinase/protein kinase B (PI3K/Akt) signaling-related proteins, such as phosphorylated PI3K (p-PI3K) and p-Akt, was observed in MI of mice.^12^ The PI3K/Akt signaling pathway is related to myocyte hypertrophy and collagen synthesis in primary neonatal cardiomyocytes.^13^ In this study, we aimed to explore whether vaspin could attenuate heart remodeling through blocking PI3K/Akt pathway.

There is a connection between oxidative stress, a stimulant for the signal transduction in cardiac cell pathological conditions,^14^ and the pathogenesis and occurrence of ischemic heart disease. Oxidative stress plays a vital role in disorders related to vascular structure and function.^15^ Dysfunction of cardiac mitochondria, a hallmark of heart failure and a major cause of oxidative stress, damages cellular components and thus forms a vicious circle.^16^ This study was designed to determine whether vaspin could alleviate cardiac remodeling through inhibiting PI3K/Akt pathway to relieve oxidative stress.

## MATERIAL AND METHODS

### Animals

Male Sprague–Dawley (SD) rats (160–180 g) from Vital River Biological Co, Ltd (Beijing, China) were housed in an environment of 22 ± 1°C, a relative humidity of 40%–60%, a 12-hour light/dark cycle, and with free access to standard chow and tap water. The animal experiment was consistent with the regulations and guidelines of Medical School of Southeast University institutional animal care and performed based on the Association for Assessment and Accreditation of Laboratory Animal Care and the Institutional Animal Care and Use Committee guidelines (NIH publication, revised in 2016).

### Myocardial Infarction Model

MI was induced by coronary artery ligation (CAL) with sterile techniques in rats as previously described.^17^ The rats were randomized to receive either the left anterior descending CAL or sham operation (sham group) under isoflurane (2.5%–3.0%) anesthesia. The heart was exposed using a left intercostal thoracotomy, and the left coronary artery was looped by a single nylon suture (7-0). Then, the heart was promptly repositioned into the chest. The sham group was treated the same way except that CAL was not performed. Rats were administered vaspin (320-ng/kg body weight; PeproTech, NJ) every day by intraperitoneal (i.p.) injection for 4 weeks.

### Transverse Aortic Constriction Model

The rats were randomized to receive either the transverse aortic constriction (TAC) or sham operation (sham group) under isoflurane (2.5%–3.0%) anesthesia. Midline sternotomy was performed, and the aorta was visualized. A 4‐0 suture was placed between the brachiocephalic artery and the left common carotid artery. The suture was tightened around a blunt 21‐gauge needle placed adjacent to the aorta. The needle was then removed, and the chest was closed. The sham group underwent the same operation but without aortic constriction. Rats were administered vaspin (320-ng/kg body weight; PeproTech) every day by i.p. injection for 4 weeks.

### Ang II Infusion Model

The rats were subjected to a 4-week infusion of Ang II (Sigma, MO) or saline (solvent control) administered by mini-osmotic pumps (model 2004; ALZET Osmotic Pumps, CA) at an infusion rate of 500 ng/kg/minute. Rats were administered vaspin (320-ng/kg body weight; PeproTech) every day by i.p. injection for 4 weeks.

### Echocardiography

Transthoracic echocardiography was conducted using an ultrasound system (VisualSonics, Toronto, Canada) with a 21-MHz probe under isoflurane (2.5%–3.0%) anesthesia. Measurements over 3 consecutive cardiac cycles were averaged. The ejection fraction (EF) and fractional shortening (FS) of left ventricular (LV) of rats were calculated. The LV diameter in diastole, LV diameter in systole, LV volumes in systole (LVVs), and LV volumes in diastole (LVVd) were measured.

### Hemodynamic Monitoring

Under isoflurane (2.5%–3.0%) anesthesia, insertion of a conductance micromanometer catheter (1.4F; Millar Instruments, TX) was made into the LV chamber through the left carotid artery for hemodynamic monitoring. The maximum of the first differentiation of LV pressure (LV +dp/dt_max_), LV systolic pressure (LVSP), and LV end-diastolic pressure (LVEDP) was measured through the PowerLab data acquisition system (AD Instruments, Sydney, Australia).

### Masson Staining

Masson staining (Service Biological Technology Co, Ltd, Wuhan, China) was used to evaluate cardiac fibrosis. Three to 5 fields (about 30–50 cells per field) were randomly chosen from each of the 3 cardiac sections (5 µm) from each rat and analyzed using a light microscope (Carl Zeiss GmbH, Oberkochen, Germany). Image-Pro Plus software (Media Cybernetics, Inc, MD) was used to analyze the images.

### Culture of Neonatal Rat Cardiomyocytes

Primary cardiomyocytes were isolated from 1- to 2-day-old newborn SD rats (Vital River Biological Co). Hearts were excised and digested in PBS with collagenase type II (Worthington Biochemical Corp, NJ) and pancreatin (Sigma). After discarding the atria and great vessels, the ventricles were cut into small pieces and further digested with collagenase type II and pancreatin. The neonatal rat cardiomyocytes (NRCMs) from digestion were collected and cultured in complete Dulbecco's modified Eagle's medium (Gibco, Invitrogen Inc) for 2–4 hours to reduce fibroblasts and enrich for cardiomyocytes. The NRCMs were cultured at 37°C with 5% CO_2_. Vaspin (100 ng/mL) and Ang II (10^−6^ M; Sigma) were devolved in PBS and added into the growth media in 6-cm plates (10^5^ cells/plate) for 24 hours. Wortmannin (100 nM; Selleck, Shanghai, China) and MK2206 (5 mM; Selleck) were added into the growth media.

### Culture of Neonatal Rat Cardiac Fibroblasts

Neonatal rat cardiac fibroblasts (NRCFs) from 1- to 2-day-old SD rats were isolated from cardiomyocytes by gravity separation and grown to confluence on 10-cm cell culture dishes with Dulbecco's modified Eagle's medium containing 10% FBS, 1% penicillin, and 1% streptomycin at 37°C in humid air with 5% CO_2_ and 95% O_2_. CFs from the second passage were used for the subsequent experiments. Vaspin (100 ng/mL) and Ang II (10^−6^ M) were devolved in PBS and added into the growth media in 6-cm plates (10^5^ cells/plate) for 24 hours.

### PI3K/Akt and Nox1 Overexpression

Adenovirus carrying PI3K, Akt, or NADPH oxidases 1 (Nox1) coding sequence (GeneChem, Shanghai, China) was diluted in PBS and added into the media. Adenovirus carrying green fluorescent protein was used as control. NRCMs or NRCFs were infected with adenovirus at 50 multiplicity of infection for 24 hours.

### Quantitative Real-Time Polymerase Chain Reaction (qRT-PCR)

The rats were sacrificed by pentobarbital overdose (100 mg/kg, i.p.), and the hearts were harvested. Total RNA was extracted using TRIzol (Ambion, TX). The cDNA was extracted from the RNA by reverse transcription using 10-μL random primers (Genscript, Nanjing, China; Table 1) following the instructions of the PrimeScript RT Master Mix (TaKaRa Biomedical Technology, Beijing, China). All cDNA was stored at − 80°C before use. mRNA was determined using SYBR Green I fluorescence. All samples were amplified in triplicates for 40 cycles in a 384-well plate. The relative gene expression was calculated using the values of Δcycle threshold as a relative quantity to the endogenous control.

**TABLE 1.** List of Used Primers for qRT-PCR

Gene	Species	Forward Primer	Reverse Primer
Collagen I	Rat	TCAAGATGGTGGCCGTTAC	CTGCGGATGTTCTCAATCTG
Collagen III	Rat	CGAGATTAAAGCAAGAGGAA	GAGGCTTCTTTACATACCAC
ANP	Rat	GAGCAAATCCCGTATACAGTGC	ATCTTCTACCGGCATCTCCTCC
BNP	Rat	GCTGCTGGAGCTGATAAGAGAA	GTTCTTTTGTAGGGCCTTGGTC
GAPDH	Rat	GGCACAGTCAAGGCTGAGAATG	ATGGTGGTGAAGACGCCAGTA

GAPDH, glyceraldehyde 3-phosphate dehydrogenase; qRT-PCR, quantitative real-time polymerase chain reaction.

### Western Blotting

Heart tissues or cultured cells were sonicated in RIPA lysis buffer and homogenized. The supernatant was obtained after centrifugation at 12,000*g* for 10 minutes at 4°C and debris removal. After electrophoresis, the proteins were transferred to a nitrocellulose membrane, probed with p-PI3K (17,366, 1:1000; CST, MA), PI3K (4257, 1:1000; CST), p-Akt (13,038, 1:1000; CST), Akt (9272, 1:1000; CST), and Nox1 (ab131088, 1:2000; Abcam, MA) primary antibodies, and incubated with secondary antibodies (Abcam). The bands were visualized by enhanced chemiluminescence substrate (BioChannel Biological Technology Co, Ltd). The total protein level was normalized to the protein level of glyceraldehyde 3-phosphate dehydrogenase (BS65483M, 1:5000; Bioworld Technology Inc, MN).

### Immunofluorescence

The heart samples were then fixed with 4% paraformaldehyde, embedded in paraffin, and sectioned into 5-cm-thick slides. Then, the samples were incubated with primary antibody against 8-hydroxy-2′-deoxyguanosine (8-OHdG; sc-66036, 1:1000; Santa, TX) at 4°C overnight, followed by the corresponding secondary antibodies (Jackson ImmunoResearch, PA) for 2 hours at room temperature. Then, 4′,6-diamidino-2-phenylindole (Life Technologies Co, NY) was used to counterstain the nucleus. The images were captured using a fluorescence microscope (Carl Zeiss GmbH, Oberkochen, Germany).

### Nox Activity Measurement

Nox activity in CFs was evaluated using enhanced lucigenin chemiluminescence. The CFs were sonicated and homogenized. The supernatant was obtained after centrifugation at 12,000*g* for 10 minutes at 4°C and debris removal. NADPH (100 μM) was added to the supernatant as a substrate to react with Nox and generate superoxide anions. A microplate reader (BioTek, VT) was used to measure the light emission produced by the reaction of lucigenin (5 μM) and superoxide anions once every minute for 10 minutes. The value representing the Nox activity was expressed as the mean light units per minute per milligram of protein.

### Superoxide Anion Measurement

Superoxide anions in the CFs were measured using lucigenin-derived chemiluminescence. The CFs were sonicated and homogenized at 4°C. The supernatant was collected after centrifugation at 12,000*g* for 10 minutes at 4°C and debris removal. Dark-adapted lucigenin (5 μM) was added to the supernatant to react with superoxide anions and trigger photon emission, which was then measured using a microplate reader (BioTek) once every minute for 10 minutes. The value representing the superoxide anion level was expressed as the mean light units per minute per milligram of protein.

### Malondialdehyde Level Measurement

The CF samples were homogenized in lysis buffer (Thermo Fisher Scientific, MA). The malondialdehyde (MDA) level was determined by the ELISA kit (USCN Business Co, Ltd, Wuhan, China).

### Determination of the Superoxide Dismutase Activity

The samples were homogenized in lysis buffer (Thermo Fisher Scientific). The superoxide dismutase (SOD) activity was measured following the manufacturer's instructions (Beyotime, Shanghai, China).

### Statistical Analyses

Data were presented as mean ± SEM. Using GraphPad Prism 7.0 (GraphPad Software Inc, CA), statistical significance among groups was assessed by 1-way analysis of variance with the Bonferroni post hoc test. Statistical significance was defined as a 2-tailed *P* value <0.05.

## RESULTS

### Vaspin Alleviated Cardiac Dysfunction in MI Rats

LVSP was decreased in MI rats, and this decrease was reversed by vaspin treatment. LVEDP was elevated in MI rats, which was reduced after vaspin administration. LV +dp/dt_max_ (mm Hg/s) was decreased in MI rats, and the decrease of LV +dp/dt_max_ in MI rats was elevated after vaspin treatment. The elevated LVVd and LVVs and decreased EF and FS in MI rats were reversed by vaspin treatment (Table 2).

**TABLE 2.** Echocardiographic Examination of the Left Ventricular Function

Variables	Sham + Saline	Sham + Vaspin	MI + Saline	MI + Vaspin
LVSP (mm Hg)	134.9 ± 5.7	135.4 ± 5.3	116.5 ± 3.4*	129.6 ± 3.5†
LVEDP (mm Hg)	0.3 ± 0.7	0.4 ± 0.6	16.5 ± 1.2*	5.5 ± 0.6†
LV +dp/dtmax (mm Hg/s)	3648.5 ± 116.6	3586.4 ± 105.4	1985.7 ± 99.5*	2796.5 ± 126.6†
LVVd (µL)	125.0 ± 9.0	122.6 ± 9.3	204.4 ± 10.8*	141.4 ± 9.6†
LVVs (µL)	46.2 ± 2.8	45.9 ± 3.6	123.6 ± 11.5*	68.4 ± 3.3†
LVIDd	7.5 ± 0.2	7.6 ± 0.3	12.4 ± 0.7*	9.5 ± 0.3†
LVIDs	4.7 ± 0.2	4.7 ± 0.2	9.6 ± 0.5*	6.4 ± 0.3†
EF (%)	63.1 ± 2.2	62.6 ± 1.3	39.6 ± 5.0*	51.6 ± 3.6†
FS (%)	37.4 ± 3.7	37.7 ± 4.7	22.6 ± 2.8*	32.7 ± 2.5†

The results are expressed as the mean ± SEM of 8 rats per group.

* *P* < 0.05 versus the Sham + Saline group.

† *P* <0.05 versus the MI + Saline group.

LVIDd, LV diameter in diastole; LVIDs, LV diameter in systole.

### Vaspin Alleviated Cardiac Remodeling in Rats

**FIGURE 1.**
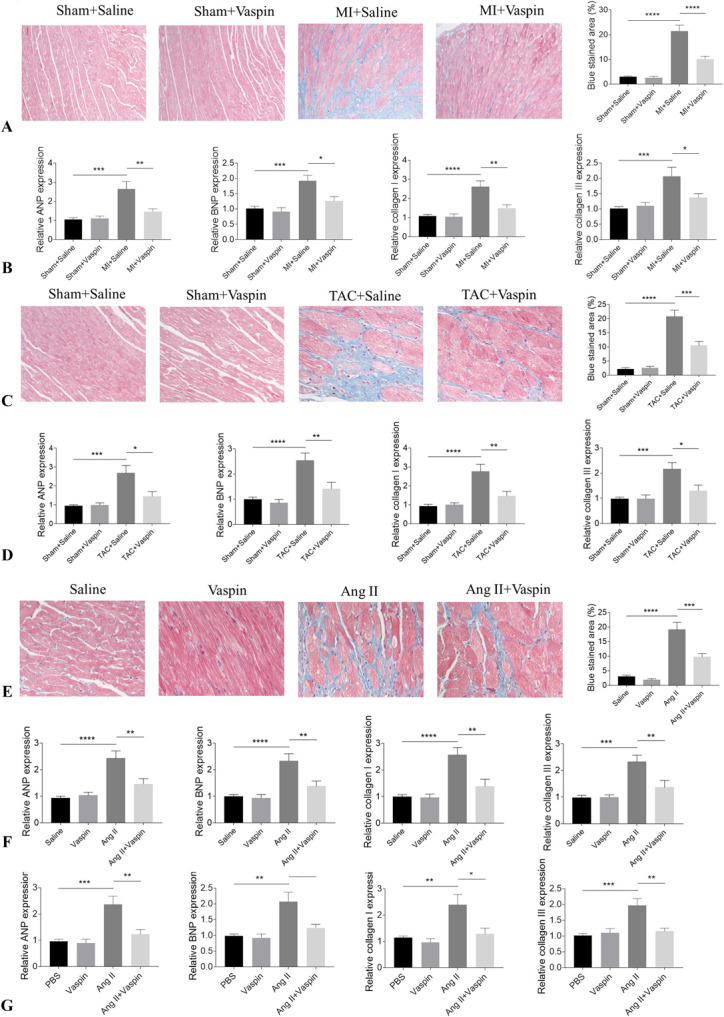
Vaspin alleviated cardiac remodeling of rats. A, The increase of cardiac fibrosis was alleviated by vaspin in MI rats. B, The increases of ANP, BNP, collagen I, and collagen III in the MI rat heart were inhibited by vaspin. C, The increase of cardiac fibrosis was alleviated by vaspin in TAC rats. D, The increases of ANP, BNP, collagen I, and collagen III in the TAC rat heart were inhibited by vaspin. E, The increase of cardiac fibrosis was alleviated by vaspin in Ang II-treated rats. F, The increases of ANP, BNP, collagen I, and collagen III in the Ang II-treated heart were inhibited by vaspin. G, The Ang II-induced increases of ANP and BNP in NRCMs and increases of collagen I and collagen III in NRCFs were inhibited by vaspin. The results are expressed as mean ± SEM. n = 8 for each group. *P < 0.05, **P < 0.01, ***P < 0.001, and ****P < 0.0001.

The cardiac fibrosis was increased in MI rats, which was attenuated by vaspin administration (Fig. 1A). The increased expression levels of ANP, BNP, collagen I, and collagen III in the MI rat heart were attenuated by vaspin administration (Fig. 1B). The cardiac fibrosis was increased in TAC rats, which was attenuated by vaspin administration (Fig. 1C). The increased expression levels of ANP, BNP, collagen I, and collagen III in the TAC rat heart were attenuated by vaspin administration (Fig. 1D). The cardiac fibrosis was increased in Ang II-treated rats, which was attenuated by vaspin administration (Fig. 1E). The increased expression levels of ANP, BNP, collagen I, and collagen III in the heart of Ang II-treated rats were attenuated by vaspin administration (Fig. 1F). ANP and BNP levels rose in Ang II-treated NRCMs, and these increases were inhibited by vaspin treatment. The levels of collagen I and collagen III were increased in NRCFs, which was alleviated after vaspin treatment (Fig. 1G).

### Vaspin Inhibited the Ang II-Induced Enhancement of PI3K/Akt Pathway

**FIGURE 2.**
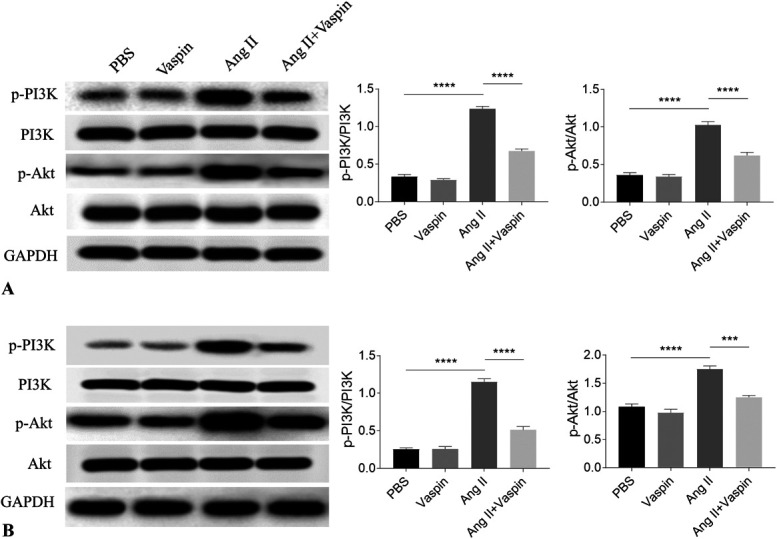
Vaspin inhibited the enhancement of PI3K/Akt pathway. A, The Ang II-induced increases of p-PI3K and p-Akt were inhibited by vaspin in NRCMs. B, The Ang II-induced increases of p-PI3K and p-Akt were inhibited by vaspin in NRCFs. The results are expressed as mean ± SEM. n = 4 for each group. *P < 0.05, **P < 0.01, ***P < 0.001, and ****P < 0.0001.

The p-PI3K and p-Akt levels were elevated in Ang II-treated NRCMs, which was inhibited by vaspin (Fig. 2A). Ang II treatment induced the increases of p-PI3K and p-Akt in NRCFs, and these increases were blocked by vaspin administration (Fig. 2B).

### PI3K or Akt Overexpression Reversed the Attenuating Effects of Vaspin in NRCMs and NRCFs

**FIGURE 3.**
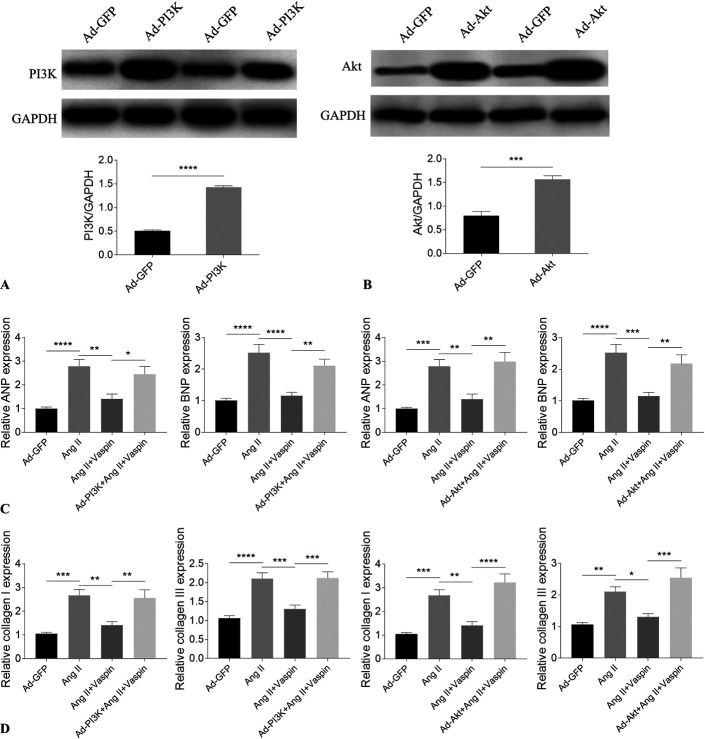
Overexpression of PI3K or Akt reversed the effects of vaspin. A, The PI3K level was increased in NRCMs after overexpression. B, The Akt level was increased in NRCMs after overexpression. C, PI3K or Akt overexpression reversed the attenuating effects of vaspin on the Ang II-induced increases of ANP and BNP in NRCMs. D, PI3K or Akt overexpression reversed the attenuating effects of vaspin on the Ang II-induced increases of collagen I and collagen III in NRCFs. The results are expressed as mean ± SEM. n = 4 for each group in A and B, and n = 8 for each group in C and D. *P < 0.05, **P < 0.01, ***P < 0.001, and ****P < 0.0001.

PI3K and Akt levels were both increased in NRCMs after overexpression (Fig. 3A, B). PI3K overexpression reversed the attenuating effects of vaspin on the increases of ANP and BNP in Ang II-treated NRCMs. The inhibiting effects of vaspin on the increases of ANP and BNP in Ang II-treated NRCMs were also reversed after Akt overexpression (Fig. 3C). PI3K overexpression reversed the blocking effects of vaspin on the increases of collagen I and collagen III in Ang II-treated NRCFs. The inhibiting effects of vaspin on the increases of collagen I and collagen III in Ang II-treated NRCFs were reversed by Akt overexpression (Fig. 3D).

### Vaspin Alleviated Oxidative Stress in MI Rats

**FIGURE 4.**
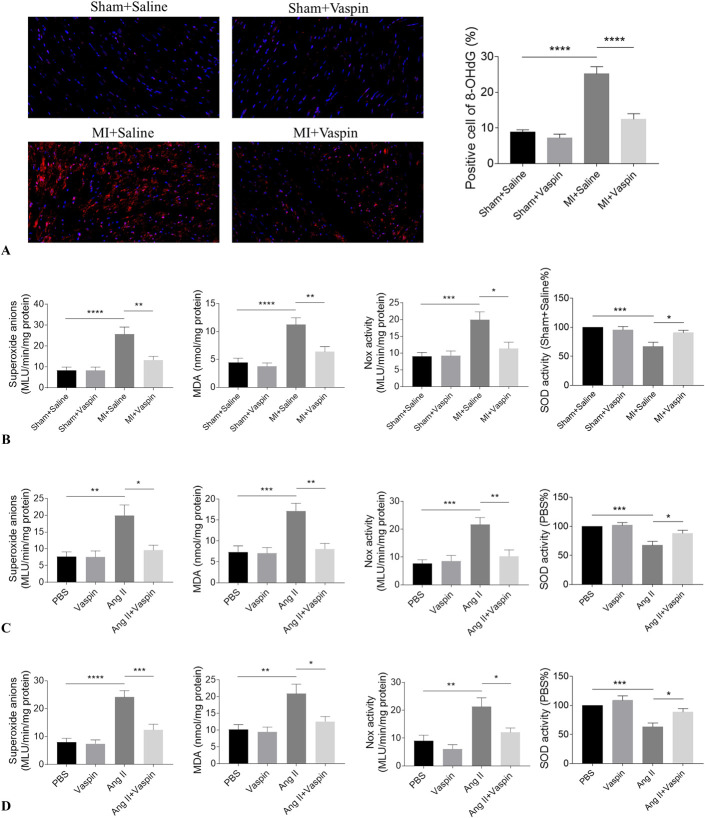
Vaspin attenuated oxidative stress. A, Vaspin inhibited the increases of 8-hydroxy-2′-deoxyguanosine positive cells in the MI rat heart. B, Vaspin reversed the increases of superoxide anions, MDA, and Nox activity and the decrease of SOD activity in the MI rat heart. C, Vaspin reversed the increases of superoxide anions, MDA, and Nox activity and the decrease of SOD activity in NRCMs. D, Vaspin reversed the increases of superoxide anions, MDA, and Nox activity and the decrease of SOD activity in NRCFs. The results are expressed as mean ± SEM. n = 8 for each group. *P < 0.05, **P < 0.01, ***P < 0.001, and ****P < 0.0001.

8-OHdG is widely used as a biomarker for oxidative stress. The number of 8-OHdG positive cells was increased in the heart of MI rats, and this increase was attenuated by administrating of vaspin (Fig. 4A). The levels of superoxide anions, MDA, and Nox activity were increased, and SOD activity was reduced in the MI rat heart, which was reversed by vaspin administration (Fig. 4B). Ang II treatment elevated the levels of superoxide anions, MDA, and Nox activity and reduced SOD activity in Ang II-treated NRCMs, which was reversed by vaspin treatment (Fig. 4C). The levels of superoxide anions, MDA, and Nox activity were increased, and SOD activity was reduced in Ang II-treated NRCFs, which was reversed by vaspin treatment (Fig. 4D).

### Nox1 Overexpression Reversed the Attenuating Effects of Vaspin in NRCMs and NRCFs

**FIGURE 5.**
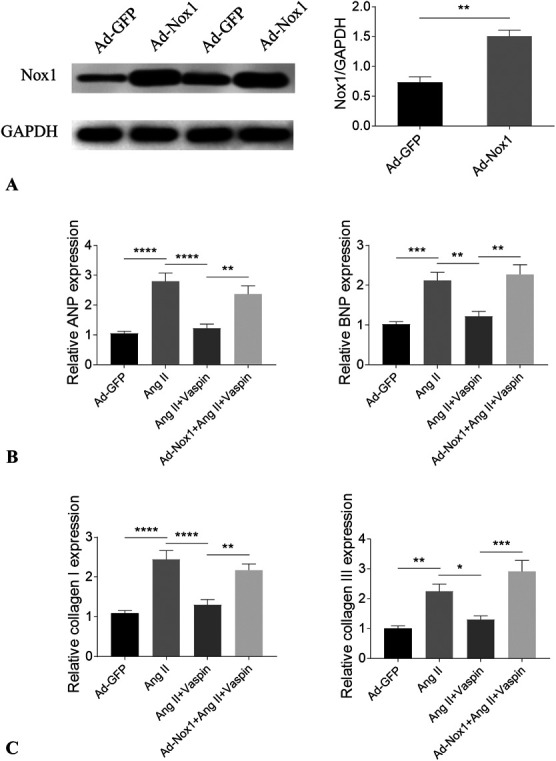
Overexpression of Nox1 reversed the effects of vaspin. A, The Nox1 level was increased in NRCMs after treatment with adenovirus carrying Nox1. B, Nox1 overexpression reversed the attenuating effects of vaspin on the Ang II-induced increases of ANP and BNP in NRCMs. C, Nox1 overexpression reversed the attenuating effects of vaspin on the Ang II-induced increases of collagen I and collagen III in NRCFs. The results are expressed as mean ± SEM. n = 8 for each group. *P < 0.05, **P < 0.01, ***P < 0.001, and ****P < 0.0001.

The Nox1 level was increased in the NRCMs after overexpression through adenovirus carrying Nox1 treatment (Fig. 5A). Nox1 overexpression reversed the attenuating effects of vaspin on the increases of ANP and BNP in Ang II-treated NRCMs (Fig. 5B). The inhibiting effects of vaspin on the increases of collagen I and collagen III in Ang II-treated NRCFs were reversed by Nox1 overexpression (Fig. 5C).

### Inhibition of PI3K/Akt Pathway Alleviated Oxidative Stress in NRCMs and NRCFs

**FIGURE 6.**
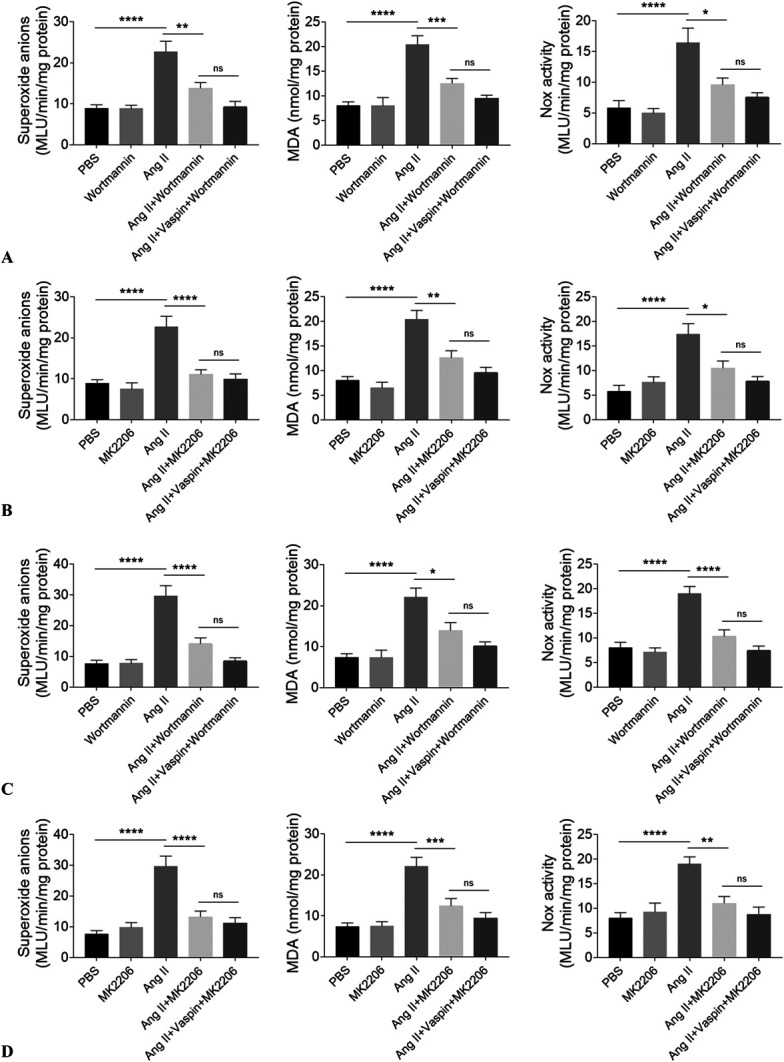
Inhibiting PI3K/Akt pathway alleviated oxidative stress. A, Treatment with wortmannin (an PI3K inhibitor) attenuated the Ang II-induced increases of superoxide anions, MDA, and Nox activity in NRCMs. B, Treatment with MK2206 (an Akt inhibitor) attenuated the Ang II-induced increases of superoxide anions, MDA, and Nox activity in NRCMs. C, Treatment with wortmannin attenuated the Ang II-induced increases of superoxide anions, MDA, and Nox activity in NRCFs. D, Treatment with MK2206 attenuated the Ang II-induced increases of superoxide anions, MDA, and Nox activity in NRCFs. The results are expressed as mean ± SEM. n = 8 for each group. ^ns^*P* > 0.05, *P < 0.05, **P < 0.01, ***P < 0.001, and ****P < 0.0001.

Wortmannin, an inhibitor of PI3K, inhibited the increases of superoxide anions, MDA, and Nox activity in Ang II-treated NRCMs (Fig. 6A). The increases of superoxide anions, MDA, and Nox activity in Ang II-treated NRCMs were attenuated after treatment with MK2206, an Akt inhibitor (Fig. 6B). Wortmannin treatment inhibited the Ang II-induced increases of superoxide anions, MDA, and Nox activity in NRCFs (Fig. 6C). The Ang II-induced increases of superoxide anions, MDA, and Nox activity in NRCFs were attenuated after MK2206 treatment (Fig. 6D).

## DISCUSSION

The following are the novel findings of this work: Vaspin alleviated cardiac fibrosis of MI, TAC, and Ang II-treated rats. The Ang II-induced fibrosis of NRCFs and hypertrophy of NRCMs were reversed by vaspin treatment. The enhanced PI3K/Akt pathway and oxidative stress were inhibited after vaspin treatment. The Ang II-induced enhancement of oxidative stress was attenuated by treatment with PI3K or Akt inhibitor in NRCMs and NRCFs. Vaspin attenuated Ang II-induced hypertrophy of NRCMs and fibrosis of NRCFs through blocking PI3K/Akt pathway to alleviate oxidative stress.

Post‐MI remodeling deteriorates in case of a larger infarct size, myocardial hemorrhage, and microvascular obstruction.^18,19^ Almost half of the patients demonstrate LV remodeling after MI, and the patients with LV remodeling have a higher risk of hospitalization for heart failure than those without it.^20^ Serum vaspin, a significant prognostic marker of major adverse cardiac events in acute MI,^8^ functions as an adipokine to inhibit myocardial apoptosis and thus to attenuate cardiac ischemia/reperfusion (I/R) injury.^21^ We presently found that LVSP, LVEDP, LV +dp/dt_max_ (mm Hg/s), EF, FS, LV diameter in diastole, LV diameter in systole, LVVs, and LVVd were increased in MI rats, and these increases were inhibited by vaspin treatment. These results indicate that vaspin can improve the cardiac dysfunction of heart failure. The increases of ANP, BNP, collagen I, and collagen III were attenuated by vaspin in the heart of MI, TAC, and Ang II-treated rats. In addition, the Ang II-induced increases of ANP and BNP in NRCMs and the Ang II-induced increases of collagen I and collagen III in NRCFs were suppressed by vaspin treatment. The above results illustrate that vaspin can alleviate cardiac injury and fibrosis in heart failure.

Akt was enhanced in the Ang II-stimulated hypertrophy of cardiomyocytes^22^ and fibrosis of fibroblasts.^23^ The signaling pathway of PI3K/Akt was also enhanced in the cardiomyocytes with hypertrophy^24,25^ and cardiac fibroblasts with fibrosis.^26^ We presently found that the PI3K/Akt pathway was enhanced in Ang II-treated NRCMs and NRCFs, which was inhibited by vaspin treatment. Overexpression of PI3K or Akt reversed the alleviating effects of vaspin on Ang II-induced hypertrophy of NRCMs and fibrosis of NRCFs. These results indicate that vaspin can attenuate cardiac remodeling (including cardiomyocyte hypertrophy and cardiac fibroblast fibrosis) through blocking the PI3K/Akt pathway.

Normally, a small amount of ROS is produced and quickly undergoes dismutation to hydrogen peroxide by SOD. However, under some stressful conditions, excessive ROS and superoxide production can exceed the antioxidant defense ability and thus become toxic to the cell.^27^ Oxidative stress is enhanced in various cardiac diseases to cause heart remodeling.^28–30^ In our current study, we found increased oxidative stress in the MI rat heart, and vaspin administration reversed this increase. Nox1 overexpression reversed the inhibiting effects of vaspin on oxidative stress. These results demonstrate that vaspin alleviates cardiac remodeling by reducing oxidative stress. We also found that the inhibition of PI3K or Akt could reduce the oxidative stress in Ang II-treated NRCMs and NRCFs. These results indicate that vaspin attenuates cardiac remodeling by blocking PI3K/Akt pathway to alleviate oxidative stress.

In conclusion, vaspin can alleviate cardiac dysfunction in MI and attenuate cardiac fibrosis. The pathway of PI3K/Akt and oxidative stress are enhanced in hypertrophic cardiomyocytes and fibrotic cardiac fibroblasts. Vaspin alleviates cardiac remodeling by inhibiting PI3K/Akt pathway to reduce oxidative stress.
